# Motives for khat use and abstinence in Yemen - a gender perspective

**DOI:** 10.1186/1471-2458-10-735

**Published:** 2010-11-27

**Authors:** Felix Wedegaertner, Hussein al-Warith, Thomas Hillemacher, Bert te Wildt, Udo Schneider, Stefan Bleich, Dirk Breitmeier

**Affiliations:** 1 Center for Addiction Research, Department of Psychiatry, Social Psychiatry and Psychotherapy, OE 7110, Hannover Medical School, 30623 Germany; 2 Department of Psychiatry, Psychotherapy and Psychosomatics, Luebbecke Hospital, Germany; 3 Department of Forensic Medicine, Medical Faculty, University of Mainz, Germany

## Abstract

**Background:**

Khat consumption is widespread in Yemeni society and causes problems both in economic development and public health. Preventive measures have been largely unsuccessful and the cultivation continues to proliferate. The gender-specific motives for khat use and abstinence were studied to create a toe-hold for more specific interventions.

**Methods:**

In a quota sample with equal numbers of males, females, abstainers and consumers, 320 subjects were interviewed on their specific opinions about khat and its impact on subjective and public health, and on social and community functioning. Strata were compared in their acceptance and denial of opinions. Notions that could predict abstinence status or gender were identified with multivariate logistic regression analysis.

**Results:**

Male khat users had a strong identification with khat use, while females were more ambivalent. The notion that khat consumption is a bad habit (odds ratio (OR) 3.4; p < 0.001) and consumers are malnuorished (OR 2.2; p = 0.046) were associated with female gender among khat users. Among the females worries about health impact (OR 3.2; p = 0.040) and loss of esteem in the family (OR 3.1; p = 0.048) when using khat predicted abstinence. Male abstainers opposed khat users in the belief that khat is the cause of social problems (OR 5.1, p < 0.001). Logistic regression reached an accuracy of 75 and 73% for the prediction of abstinence and 71% for gender among consumers. (All models p < 0.001.)

**Conclusions:**

Distinct beliefs allow a differentiation between males, females, khat users and abstainers when targeting preventive measures. In accordance to their specific values female khat users are most ambivalent towards their habit. Positive opinions scored lower than expected in the consumers. This finding creates a strong toe-hold for gender-specific public health interventions.

## Background

Khat (qat, kat) use is widespread in Yemeni society and causes problems both in economic development and public health [1]. The leaves of the Khat shrub or tree contain stimulating alkaloids, mainly cathinone (ß-ketoamphetamine), cathine ((+)-norpseudoephedrine), which are controlled substances in all western countries [2]. Between 1970 and 2000 the area devoted to khat cultivation ballooned from 8000 to 103 000 hectares [3]. Khat farming and marketing has become a large branch of Yemeni economy and a source of income for many who would otherwise be jobless. As fertile areas are scarce, the high demand for khat leaves has sparked fierce competition for water and land at the expense of traditional staple and cash crops. All water resources are currently exploited beyond the level of recharge, as 40% of all potable water in Yemen is used in khat cultivation [4]. Strong population growth and declining crude oil production rates have added to the country's problems [5].

Khat use is widespread in some parts of Yemen, even among children. The prevalence has been estimated at 80% for males and 50% for females in the capital Sana'a at age fifteen and above [6]. Between 15-20% of children under age 12 are also daily consumers [7]. For consumers, much of the day is spent buying and chewing khat, severely affecting working hours and thus family income [8]. Khat has been used traditionally in mainly male social rituals, get-togethers lasting for hours creating cultural identity, even among immigrants [9]. But khat is also used by workers to reduce feelings of fatigue.

The desired effect can only be achieved when chewing the fresh younger leaves, as the main psychoactive constituent is degraded as the leaves wilt [10]. Therefore, khat habits have remained in those areas where the plant has been indigenous for centuries. Khat rituals are lengthy and can take up to twelve hours. Chewing the leaves makes people feel more alert, euphoric and talkative, and suppresses appetite [11,12]. Regular khat use is associated with a rise in arterial blood pressure and heart rate, corresponding to levels of cathinone in the plasma [13]. A multitude of psychiatric and somatic symptoms, such as Insomnia and affective symptoms, anorexia, gastric disorders, depression, liver damage and cardiac complications have been described in regular khat users[8]. In spite of this, a previous study showed no adverse psychological symptoms in khat users as measured by the SCL-90 [14,15] and most khat users describe mainly pleasurable experiences.

Cultivation has increased sharply over the past decades in many countries [16,17], as khat has become a globally traded commodity. Despite the fact that cathinone being a Schedule I and cathine being Schedule III substances internationally, Khat as a plant in itself (unlike coca leaves for example) is not under international control, which is one of the reason why its cultivation is still hard to control. Fresh leaves are smuggled by air to the USA and Europe, selling for 300-500 USD a kilo [18].

In recent years, the Yemeni government have recognized the harmful effects of the khat complex on community functioning. So far prevention programs have been largely unsuccessful [7,19]. One reason for this may be half-heartedness in the approach, as some policymakers fear that irradication from the market would cause a possible diversion to use more harmful substances. In its report 2007, the World Bank stated that "research on the causes and consequences of khat consumption, and the behavioural pressures associated with khat uptake is limited in number and scope" and encouraged "research activities into these issues to refine the targeting of interventions for maximum effect" [7]. While excellent reviews about khat in general have been published [1,7,8,11,20], no reports address motives for khat consumption and abstinence, many are either anecdotic, focus too little on the social aspects of the drug or pertain to different cultures [21-24]. It was the aim of this study to address these issues and gain a deeper understanding of the gender-specific motivation for khat use or abstinence and the believed effects, as we suspected that these sustain khat habits in Yemeni society above and beyond khat's addictive potential.

## Methods

### Participants

For the purpose of this study, a semi-rural khat growing area in the Yemeni highlands was sought out. Ideally suited was the Wadi Dhar, located about 15 km north west of the capital Sana'a. A fertile valley dotted with oases and small villages, it is an important khat production area. Subjects were approached separately at male and female social gathering places by a Yemeni interviewer. They were recruited consecutively during a period of three months in 2007. Participation was optional and involved no incentive. Subjects had to be at least fourteen years old. As khat consumption is unevenly distributed between genders a quota sampling strategy was applied. With the first two screening questions, they were placed in one of four equal strata according to khat consumption (active consumers vs. long-term khat abstainers) and gender. Recently abstinent former khat consumers were excluded. Data acquisition for each one of the four strata was closed after 80 persons had been interviewed. Recruitment was aborted when it turned out that the corresponding stratum was already closed at that stage of the data collection process. In total, 320 persons were interviewed.

### Interview

As a standardised method for surveying opinions about khat is lacking, the items were constructed from hypotheses stated in the pertinent literature [1,3,8,12,13,15,22-27] and popular beliefs. The interview was pre-tested with twenty subjects, which led to an expansion of the number of items. Frequency, duration and khat consumption years were collected if applicable. Also marital status, age, education, vocational training, occupation, and primary source of income were requested. The main part of the interview consisted of 57 questions pertaining to positive and negative beliefs about khat in general, the physical effects and the effects on mentality and social functioning of consumers. The order of questions was randomised. The interview was conducted face-to-face - orally for the illiterate - in Arabic, using a standardised data collection form. A translated list of all questions is reproduced in Additional File 1. Four alternative answers were permitted "yes", "partly", "no" and "unsure/no response".

### Statistics

Statistical analysis was conducted using the Statistical Package for the Social Sciences Version 18.0. An approval rating for the individual items was calculated for the respective strata. The three possible answer alternatives were assumed to be on a linear scale. An approval rating of 100% corresponded to all subjects answering "yes" on the respective item. Consequently, a rating of 0% corresponded to all subjects answering "no". This scale was split in three segments of equal size. Items were regarded as accepted when the approval rating was 66.7% or above. They were regarded as rejected at approval ratings of 33.3 and below. By applying this method, those subjects in favour of a notion had to outnumber those opposing it by more than two to one (and vice versa), while not being outnumbered by the same ratio by those who only partly agreed. Otherwise the item had to be regarded as undecided. In a second step, opinions were tested between strata using the Mann-Whitney U-test for items on an ordinal scale, in order to find those items where approval rating differed significantly between females and males, consumers and abstainers. These were consequently referred to as polarizing items.

The analysis was restricted to four predefined configurations: male consumers vs. male abstainers, female consumers vs. female abstainers, male abstainers vs. female abstainers, male consumers vs. female consumers. In addition to p-values z-scores were indicated at z > 3.0, which was equivalent to a two-sided statistical significance of p < 0.0027. Raw differences in acceptance between strata were represented by percentage points (pp).

### Logistic regression analysis

In a third step, logistic regression analysis was used to determine whether items that showed statistical significance in univariate analysis could correctly predict abstinence and female gender. For methodological reasons, only those items that had been answered by at least 95% of the respective 160 subjects were included. Covariates were included using stepwise forward inclusion by likelihood ratio, in order to apply a multivariate filter for those items with the biggest predictive value.

## Results

### Description of the sample

Roughly 500 subjects were contacted during recruitment, resulting in a response rate of 64%. Abstinent females were somewhat younger than the rest of the sample (Table 1). Other than that, the subsamples were comparably structured. Almost all subjects had some sort of schooling (95%), and those who had received vocational training or college education were predominantly male (among consumers: 51% vs. 33%, abstainers: 62% vs. 34%). Subjects were reluctant to disclose their occupation, the highest non-responder rate being 53% among abstinent females. Still, most subjects seem to follow a regular job or daily routine. The majority of male subjects support themselves by their own work (70% among consumers and 60% among abstainers), while most females are supported by relatives (50% among consumers and 69% among abstainers).

**Table 1:** Descriptive statistics of the sample

		Khat Consumer		Abstinent		
		
		male n = 80	female n = 80	male n = 80	female n = 80	All n = 320
Age	minimum	16	15	14	15	14
	
	median	26.0	25.0	24.0	21.5	24.5
	
	average	28.2	29.8	27.2	23.0	27.0
	
	maximum	57	70	65	48	70

Marital status	single	46%	36%	65%	73%	55%
	
	married	50%	51%	34%	25%	40%
	
	divorced		4%			0.9%
	
	widowed				1%	0.3%
	
	unknown	4%	9%	1%	1%	3.8%

Education	No school or primary school only	19%	31%	9%	19%	18.8%
	
	secondary school	28%	34%	30%	46%	34.4%
	
	vocational training or college	51%	33%	62%	34%	45.0%
	
	unknown	3%	4%	0%	1%	1.9%

Occupation	full time	63%	34%	46%	28%	42.5%
	
	homemaker		33%	0%	19%	12.8%
	
	part time	8%	3%	14%	1%	6.3%
	
	no work	9%	1%	3%		3.1%
	
	other		5%	3%		1.9%
	
	unknown	21%	25%	35%	53%	33.4%

Primary source of income	own work	70%	36%	60%	29%	48.8%
	
	supported by relatives	9%	50%	35%	69%	40.6%
	
	pension		1%	3%		0.9%
	
	own assets	1%	3%	1%		1.3%
	
	welfare	1%				0.3%
	
	other	3%				0.6%
	
	unknown	16%	10%	1%	3%	7.5%

Khat consumption frequency	daily	79%	30%			55%
	
	at least weekly	15%	25%			20%
	
	less than weekly	6%	45%			25%
	
	unknown	0%	0%			0%
	
Length of khat ritual	[hours; avg. (min-max)]	5.4 (2-12)	4.0 (1-9)			4.7 (1-12)
	
Years of khat	[years; avg. (min-max)]	10.3 (1-30)	6.8 (1-50)			8.6 (1-50)

### Khat consumption

79% of male subjects who consume khat do this on a daily basis. Females seem to have a less intensive consumption pattern, as the majority consume khat on a "less than weekly" basis. Khat rituals, when performed, were longer among males (5.4 vs. 4.0 hours), and consuming males had used khat for longer in their lives than females at the time of the study (10.3 vs. 6.8 years). On average, daily users spend 5.3 hours (2 to 12) chewing khat. These heavy users have been on khat for an average of 10.4 years (1 to 50).

### Survey outcome

Accepted and rejected beliefs about khat are shown in Table 2. In order to get a better understanding of answering patterns the items are sorted in declining order by acceptance rate in the stratum of female khat abstainers, as these had the strongest opinions. It can immediately be seen that answering patterns of male abstainers and female subjects were almost identical while male khat users answered differently Male consumers believe in positive physical effects of khat, while strongly rejecting negative social effects. They are undecided about the majority of negative beliefs. Female consumers were less positive about their habit showing an answering pattern relatively close to that of abstainers. They also had such weak opinions about negative beliefs, that none was rejected outright. Apart from a belief that females should not consume khat, which is particular to the male stratum, abstainers of both genders did not differ substantially in their answering patterns, showing very strong negative opinions about khat and their strong rejection of the claim that khat consumption leads to stress relief. The social aspect ("improves relationships in our society") is rejected slightly less strongly by male abstainers than female abstainers.

**Table 2:** Full list of items, responses and answer rates - sorted by statement acceptance by female khat abstainers

Do you believe that...	Response rate*; inclusion regression analysis**		Accepted and rejected beliefs about khat and acceptance rates***							
			
			Consumers		Abstainers		Consumers		Abstainers	
			
			Male	Female	Male	Female	Male	Female	Male	Female
khat causes poverty and squalor in Yemeni society?			+	+	+	++	69,2%	77,4%	86,5%	**94,7%**

khat worsens oral hygiene?		F	++	++	++	++	**77,8%**	**83,8%**	**90,3%**	**93,4%**

consuming khat leads to a loss of esteem in the family?	**>**	M;F;C;A	+	+	+	++	67,1%	70,8%	87,3%	**93,0%**

khat consumption is bad for one's health?	**>**	M;F;A		+	+	++	57,8%	72,3%	82,1%	**93,0%**

khat consumption leads to loss of valuable work time?	**>**	M;F;C;A		++	++	++	65,6%	**78,7%**	**89,9%**	**92,2%**

khat causes loss of appetite?	**>**	M;F;C;A		++	++	+	63,1%	**83,8%**	**88,2%**	91,9%

khat causes a reduced need for sleep?	**>**	F;C;A	+	+	++	+	74,0%	77,6%	**88,2%**	91,8%

khat consumers are undernourished?		C		++	+	+	57,9%	**78,9%**	86,7%	91,3%

khat consumption is a bad habit and should be eliminated?	**>**	M;F;C;A		+	+	+	47,4%	77,3%	83,5%	90,3%

khat causes constipation?	**<**	C		+	+	+	65,8%	77,0%	87,3%	88,7%

khat consumption is harmful to people's esteem and sophistication?	**>**	M;F;C;A		+	+	+	58,0%	75,0%	85,3%	87,8%

khat is harmful to the unborn child?	**<**		++	+	+	+	**77,0%**	75,7%	86,2%	87,7%

khat consumption at work causes laziness?					+	+	55,9%	66,4%	80,7%	86,5%

khat leads to an unhealthy complexion?	**<**			++	+	+	59,6%	**79,2%**	74,6%	86,3%

khat consumption by mothers causes neglect of their children and the household?				+	+	+	63,9%	71,2%	80,3%	84,9%

khat causes moodyness?		C		+	+	+	66,0%	74,3%	79,5%	84,9%

khat consumption impairs the function of the jaw muscles?	**<**			+	+	+	61,2%	71,4%	85,5%	83,6%

khat rituals cause back injuries through long periods of sitting?	**<**			+	+	+	61,4%	70,3%	84,9%	83,6%

women should not consume khat, because this is especially bad for the family?			+	+	++	+	74,0%	69,6%	**89,7%**	83,3%

khat consumption causes lack of food in the family?	**>**	M;F;C;A		+	+	+	48,0%	69,6%	84,4%	82,5%

khat causes restlessness and nervousness?		C			+	+	55,8%	59,1%	80,6%	81,6%

khat leads to a change in psyche and behaviour?		C		+	+	+	55,2%	67,3%	81,8%	80,9%

the family as a whole suffers financially if khat is consumed by the adults?	**>**	M;F;C;A		+	+	+	57,6%	75,3%	82,3%	80,4%

khat consumption causes haemorrhoids?	**<**			+	+	+	47,3%	68,3%	78,4%	80,0%

khat is a reason for most family problems?			- -		+	+	***33,3%***	58,0%	73,9%	79,7%

khat houses are a source of epidemic infections?	**<**		-			+	*30,9%*	62,1%	66,4%	79,7%

khat consumption of parents leads to conduct disorders in their children?		F		+	+	+	57,4%	73,0%	86,0%	79,5%

khat consumption causes agitation and anxiousness?		C			+	+	55,1%	64,0%	78,6%	79,2%

khat is stimulating?	**>**	F;C;A	++	+	+	+	**86,9%**	73,4%	80,9%	78,6%

khat consumption is the cause of social problems?	**>**	M;C;A			+	+	41,7%	64,5%	79,2%	78,4%

khat consumers have a poor physical appearance?	**>**	M;F;C;A			+	+	38,3%	65,8%	80,0%	76,0%

khat causes impaired mood?		C			+	+	46,8%	54,6%	66,9%	75,7%

khat consumption impairs the relationships between parents and their children?					+	+	39,3%	59,3%	76,0%	73,9%

partner's ability to take care of the family is impaired by consuming khat?	**>**	M;F;C;A			+	+	51,9%	66,0%	76,3%	73,4%

children of khat consuming parents do worse in school?					+	+	44,5%	62,0%	73,0%	73,3%

khat consumers wake up tired the next morning?	**<**			+	+	+	52,7%	68,7%	75,8%	72,7%

khat causes high blood pressure?	**<**				+	+	53,6%	62,0%	70,7%	71,6%

khat is addictive?		M			+	+	33,8%	60,1%	70,7%	71,5%

khat consumption prevents the use of other (illicit) drugs?			+	+	+	+	71,5%	67,6%	67,1%	70,1%

khat causes partners to fall out with each other?		C	- -		+	+	***27,6%***	52,6%	68,0%	69,3%

khat causes depression?		C			+	+	44,2%	60,5%	70,7%	68,7%

khat causes panic attacks?	**<**				+	+	41,1%	46,5%	71,1%	68,5%

khat causes low blood sugar?	**<**			+	+	+	67,0%	72,7%	73,4%	68,1%

khat consumption is a factor in divorces?	**<**		- -				***26,3%***	50,0%	64,2%	66,0%

khat is a drug?	**<**		- -				***30,1%***	44,0%	64,6%	62,0%

khat causes loneliness?	**<**	C					36,5%	49,3%	60,3%	61,1%

khat consumption causes a higher crime rate?	**<**				+		35,3%	59,3%	68,6%	61,1%

khat causes constant aggression between spouses?	**<**		- -		+		***18,0%***	50,7%	70,6%	56,9%

khat improves memory retentiveness and concentration?		F;C	++				**74,7%**	63,9%	50,0%	48,6%

khat makes you attractively slim?	**<**				- -		37,1%	49,3%	***27,8%***	37,5%

khat rituals solve many social problems?			++				**74,0%**	50,7%	45,8%	35,1%

you feel well and relaxed while consuming khat?		C				-	55,1%	56,8%	41,2%	*32,8%*

khat rituals are the best way to spend one's time?	**>**	M;F;C;A			- -	- -	57,1%	53,3%	***32,0%***	***25,7%***

khat improves physical fitness?					- -	- -	52,9%	41,4%	***25,0%***	***18,5%***

khat improves the experience during sexual intercourse?	**<**					- -	41,9%	44,1%	44,1%	***16,4%***

khat consumption improves relationships in our society?		F			- -	- -	39,6%	34,9%	***29,4%***	***10,5%***

consuming khat leads to relief of social and mental stress?					- -	- -	46,6%	55,2%	***32,1%***	***9,9%***

* " > " = more than 95%, " < " = less than 90%

** included in logistic regression for the following subgroups: M - males (abstainers vs. consumers), F - females (abstainers vs. consumers), C - consumers (males vs. females), A - abstainers (males vs. females)

*** ++ = top 5 most strongly accepted beliefs, + = accepted beliefs, -- top 5 most strongly rejected beliefs, - = rejected beliefs, no mark = undecided

### Analysis of stratum differences - Polarizing items

Subjects differed significantly in many of their beliefs, depending on whether they were female or male abstainers or female or male consumers.

### Male consumers vs. abstainers

Disagreement reached statistical significance in 52 of the 57 items presented. Differences were strongest in the following items: Male abstainers feel much more strongly than abstainers that khat leads to constant aggression between spouses (+52.6 pp, z = 7.05, p < 0.001), is a reason for most family problems (+40.6 pp, z = 6.16, p < 0.001), is the cause of social problems (+37.5 pp, z = 6.15, p < 0.001), causes a poor physical appearance (+41.7 pp, z = 5.96, p < 0.001), causes partners to fall-out with each other (+40.4 pp, z = 5.76, p < 0.001), causes lack of food in the family (+36.4 pp, z = 5.65, p < 0.001), should be eliminated (+36.2 pp, z = 5.61, p < 0.001), is addictive (+36.9 pp, z = 5.28, p < 0.001), and leads to loss of valuable work time (+24.7 pp, z = 4.94, p < 0.001), to name only the most distinct statements.

### Female consumers vs. abstainers

Female abstainers and female consumers are slightly less pronounced in their differences, which reach statistical significance in 35 of 57 items. Female abstainers feel much more strongly than consumers that consuming khat is bad for one's health (+20.7, z = 4.47, p < 0.001), leads to a loss of esteem in the family (+22.3, z = 4.26, p < 0.001), and causes poverty (+17.7, z = 3.91, p < 0.001). Consumers say that khat improves relationships in Yemeni society (-24.34 pp, z = -4.21, p < 0.001), is the best way to spend one's time (-27.6, z = -4.17, p < 0.001), leads to relief of social and mental stress (-45.3 pp, z = -3.97, p < 0.001), and improves sexual experience during intercourse (-27.8 pp, z = -3.88, p < 0.001).

### Male and female consumers

Female and male consumers have statistically significant differences in 29 opinions about khat. Females believe much more strongly that khat consumption should be eliminated (+49.9 pp, z = 4.53, p < 0.001), leads to constant aggression between spouses (+32.7 pp, z = 4.48, p < 0.001), that khat houses are a source of epidemic infections (+31.25 pp, z = 4.42, p < 0.001), that khat causes loss of appetite (+20.6 pp, z = 4.21, p < 0.001), a poor physical appearance (+27.5 pp, z = 3.92, p < 0.001), and is addictive (+26.4 pp, z = 3.80, p < 0.001), to name only the topmost.

### Male and female abstainers

Female and male abstainers are highly consistent in their opinions and show statistically significant differences in only five of 57 items. Male abstainers believe more strongly than females that khat improves sexual experience during intercourse (+27.7 pp, z = 3.41, p < 0.001) and improves relationships in Yemeni society (+18.9 pp, z = 4.21, p < 0.001). Females believe more strongly than males that khat consumption causes poverty (-8.3 pp, z = -2.97, p = 0.003) and is bad for one's health (-11.0 pp, z = -2.62, p = 0.009)

### Logistic regression analysis

#### Males

In total, 13 covariates could be included in the analysis, as these had been answered by at least 95% of the subjects. Included items are indicated in Table 2. The logistic regression (summary Table 3) yields a statistically significant multivariate model with an accuracy of 74.8% (degrees of freedom: df = 2, coefficient of determination: R^2 ^= 0.451, p < 0.001) to predict abstinence. The model configuration indicates that males who believe that "khat is the cause of social problems" (odds ratio; OR = 5.064) and "khat consumption leads to loss of valuable work time" (OR = 3.179) are predominantly abstinent.

**Table 3:** Summary of logistic regression analysis

Males - prediction of khat abstinence (n = 115, 71.9%)					
**Predictor**	**B**	**SE**	**OR**	**CI**	**p**

is the cause of social problems	1.622	0.374	5.064	2.434-10.536	< 0.001

leads to loss of valuable work time	1.157	0.463	3.179	1.282-7.879	0.013

Constant	-4.481	0.846	0.011		

**Model Summary**					

Significance of last step			p = 0.008		

Accuracy			74.8%		

Nagelkerke's coefficient of determination			R^2 ^= 0.451		

Model significance			p < 0.001		

**Females - prediction of khat abstinence (n = 100, 62.5%)**					

Predictor	B	SE	OR	CI	p

is best way to spend time	-0.831	0.302	0.436	0.241-0.788	0.006

loss of esteem in the family	1.135	0.575	3.111	1.008-9.602	0.048

is bad for one's health	1.161	0.564	3.193	1.056-9.651	0.040

Constant	-0.962	1.185	0.382		

**Model Summary**					

Significance of last step			p = 0.031		

Accuracy			73.0%		

Nagelkerke's coefficient of determination			R^2 ^= 0.360		

Model significance			p < 0.001		

**Consumers - prediction of female gender (n = 93, 58.1%)**					

Predictor	B	SE	OR	CI	p

bad habit and should be eliminated	1.211	0.373	3.356	1.614-6.977	0.001

is best way to spend time	0.720	0.338	2.055	1.060-3.984	0.033

consumers are malnourished	0.797	0.400	2.220	1.014-4.857	0.046

Constant	-4.306	1.193	0.130		

**Model Summary**					

Significance of last step			p = 0.042		

Accuracy			71.0%		

Nagelkerke's coefficient of determination			R^2 ^= 0.314		

Model significance			p < 0.001		

**Abstainers - prediction of female gender (n = 122, 76.3%)**					

Predictor	B	SE	OR	CI	p

is bad for one's health	1.417	0.510	4.124	1.518-11.207	0.005

impairs ability to take care of the family	-0.681	0.313	0.506	0.274-0.934	0.029

Constant	-0.640	0.630	0.527		

**Model summary**					

Significance of last step			p = 0.023		

Accuracy			56.5%		

Nagelkerke's coefficient of determination			R^2 ^= 0.123		

Model significance			p = 0.003		

#### Females

A total of 17 covariates were included. The accuracy of the statistically significant multivariate model was 73.0% (df = 3, R^2 ^= 0.360, p < 0.001). Females who believe that "khat consumption leads to a loss of esteem in the family" (OR = 3.111) and "khat is bad for one's health" (OR = 3.193) are predominantly abstinent while those who believe that "chewing khat is the best way to spend one's time" (OR = 0.436) are predominantly khat users.

#### Consumers

In total, 25 covariates were included. Model accuracy was 71.0% (df = 3, R^2 ^= 0.314, p < 0.001). Consumers who believe that khat consumption "is a bad habit and should be eliminated" (OR 3.356) and "khat consumers are malnourished" (OR = 2.220) are predominantly female. Additionally, female consumers felt so strongly that "khat use is the best way to spend one's time" (OR = 2.055) that this item turned out to be statistically significant both in this model predicting gender among consumers as well as in the model explained above predicting abstinence among females.

#### Abstainers

Multivariate analysis using logistic regression yielded a model with a low accuracy of 56,0% and a low coefficient of determinance (df = 2, R^2 ^= 0.123, p = 0.003). The belief "khat is bad for one's health" (OR 4.124) predicted female gender. The belief "partner's ability to take care of the family is impaired by consuming khat" predicted male gender (OR 0.506).

## Discussion and conclusions

This study describes prevailing beliefs about the impact of khat use for both female and male consumers and abstainers and their predictive values for abstinence and gender.

It is evident that drug consumers are much less concerned about negative effects than those who abstain. This, too, is the case with khat use. E. g. most khat users deny its addictive potential. Nevertheless, certain beliefs are unanimously accepted. Among the positive beliefs, these are subjective physiological effects (reduced need for sleep, stimulation), mainly because consumers deny the negative physiological impact. Interestingly, positive social aspects of khat rituals scored relatively low in this scale, which is contrary to what one would have expected. From a broader social perspective, some negative effects are agreed upon across strata (loss of esteem in the family, worsens oral hygiene, causes poverty, harmful to the unborn child). The opinion that khat consumption prevents the use of other illicit drugs was widely accepted, echoing a cause of ambivalence about khat prevention programs in Yemen. Consequently, no stratum in this study would consistently affirm that khat is a drug. Male consumers even strongly rejected this notion. Everyone - including the female khat users - agreed that women should not consume khat. Male khat users claim that khat improves memory and concentration and that rituals actually solve social problems, but no one else is of this opinion. While abstainers agree that khat use is the cause of most family problems, leads to aggression between spouses and ultimately causes partners to fall-out with each other, the male khat users deny such beliefs. Female users were more ambivalent, as they support many negative beliefs about khat. This suggests that the identification of Yemeni men with khat use is much stronger than that of women.

In multivariate analysis, male abstainers could be differentiated from consumers by their perception of social problems stemming from khat use and the belief that khat use leads to loss of valuable work time. A typically female theme differentiating users from abstainers turned out to be health and esteem in the family. Female khat users are not very strongly of the opinion that khat use is "the best way to spend one's time", but association with female gender turned out to be strong. This was a statistically significant predictor for female gender in two multivariate analyses. In short, the economic and social impact was a strong theme separating male consumers from abstainers, while individual and family values separated female consumers from abstainers.

Women seemed to be more ambivalent towards khat. This is underscored by the fact that two negative notions were determinant for female gender (Table 3) among consumers. We found a large contrast between female khat users and abstainers on a relatively low acceptance level with regard to the belief "consuming khat is the best way to spend one's time". The question arises why this is so. It is known that compared to western societies a relatively low fraction of Yemeni women are in paid employment [28]. Most women stay with their families. There may be other relatives to care for, but that is not always the case. While women in rural areas are contributing to farm work, that's not the case in more urban regions. The Yemeni government recently recognized the fact that women underemployment is a factor hampering economic growth, however legal action toward this has only been passed in recent years. Having to fight feelings of unpleasant inactivity has much broader cultural and individual reasons and cannot be solely attributed to the economic situation. Still it may be that these are causative for the positive connotation of khat among female users.

Unsurprisingly, abstainers could not be well differentiated by the items of our interview. Univariate analysis already showed high overall agreement between genders. While the multivariate model is statistically significant, its predictive power is low. It is shown only for the sake of completeness. Nevertheless the previously mentioned result that concerns about individual health are predominantly harboured by females could be replicated, underscoring its robustness. Male khat abstainers predominantly believe that khat use of the partner impairs his ability to take care of the family. This is unsurprising as descriptive statistics already showed that male abstainers strongly feel that women should not consume khat. This also illustrates that male abstainers may attribute the role of taking care of the family stronger to females than vice versa.

### Assessment of the sample

During sampling only 64% of those subjects requested to participate actually did, which might have caused an underrepresentation of strong khat users. It may also be that females with very traditional values were underrepresented in the sample as they might have been more likely to decline participation. Therefore the results may be slightly biased, although the authors tried to minimize these effects by quota sampling. Prominent differences between genders were revealed in the demographic characteristics of occupation and source of income. The authors believe that this simply represents the current Yemeni gender roles. It may be warranted to adjust for these, should the study be repeated with a larger sample size and a stronger focus on the role of females in the Yemeni work force.

### Assessment of the constructed scale

During analysis we found that many scientific findings and a number of popular beliefs are widespread in the sample. These were the ten items, which could be included in all four logistic regression analyses as they were answered by more than 95% of all subjects. However not only should the items be answerable by almost all subjects, they should also be able to differentiate between consumers and abstainers. Many items in the lower rows of table 2 had to be regarded as undecided and most of these were popular beliefs added in the pretest. Their predictive ability turned out to be mostly low. The three items "khat consumption is a fector in divorces", "... is a drug", "... causes loneliness" had a low overall response rate mainly because of low response rates among the abstainers. Khat consumers had strong opinions about these items. The same is the case for the item "... leads to constant aggression between spouses". Females had a low response rate, but male khat users strongly opposed the notion while male abstainers were strongly in favour of it. These items may have a better predictive value than expected from the descriptive statistics, but could not be included in multivariate analysis for methodological reasons. Unsurprisingly, those items answered by most subjects addressed only a few issues of significant importance. These were the impact of khat on society and economy and the role in the family. It may be rewarding to address these complexes more thoroughly in further studies. Using logistic regression in a stepwise approach is a good way to identify the strongest predictors for a given dependent variable, but "runners-up" in predictive power can be excluded in the process. It is likely that an entirely different variable than the first reaches strongest predictive power in the second step of optimization. This may wrongly suggest predictive weakness of important variables. Therefore it was necessary to evaluate acceptance rates univariately in parallel to multivariate statistics (See Table 2).

### Relationship to other published results

While studies about the motivation for khat use exist[6,29], motives for abstinence were previously not examined. A smaller previous study already indicated that the motives of khat consuming women may well be different from those of men [6]. The results of this study support this concept. It is known that many khat users are ambivalent and regard khat use as a 'bad habit' [7]. With this study, we showed that this is especially the case among female khat users. We also confirm that the problem is downplayed by users [23]. The positive opinions consumers had about khat may also explain why a previous study yielded negative results with regard t stress levels of khat users[14,15]. Interestingly, a large proportion of participants, women in particular, did not answer the item on occupation. While the exact reasons for this remain unclear, it is important to know that the Yemeni workforce is predominantly male. It is a widely accepted female role model to stay at home with the family. Of those women who work, most are in unpaid jobs, making "invisible economic contributions"[30], particularly in rural areas. Therefore it can be assumed that the responses did not adequately reflect female economic contribution.

### Implications for public health

This study shows that different approaches to preventive measures should be taken according to gender. Female users start khat at a later age [7] and we show that they have a different pattern of use. Their ambivalence is higher, making women a well suited first target for khat prevention. Subjects in the sample felt generally well informed about khat, but had opposing opinions. Harmful effects are mostly played down, particularly by male khat users.

There are initiatives by the Yemeni government to limit khat cultivation and use. A few aspects seem to hamper these. Most importantly, Khat is a relevant source of income for farmers and marketers. Secondly, khat use is widely accepted in Yemen, even for children, and strongly intertwines with cultural and gender identity and local customs. Khat users argue very strongly that khat rituals solve many social problems. Keeping the groups of men in mind that sit together daily while chewing khat, this may not even be a misconception. Khat prevention probably needs to address all of these aspects simultaneously to be more effective. Restriction of availability is generally a good measure in drug prevention, but may not prove feasible in the case of khat in Yemen. It is the hope of the authors that the data presented in this study will be useful for prevention programs.

### Implications for further study

This study only scratches the surface of the problem of demand-side khat prevention. Previous research has shown that women start khat at a later age than males. Also, it is known that once consumption has begun it continues in most cases until old age [7]. We show that female khat users are most ambivalent. Therefore, it is suggested that research into the prevention of first khat use in children and females should be addressed next. More research is also needed to identify appropriate supply-side measures.

## Competing interests

The authors declare that they have no competing interests.

## Authors' contributions

FW performed the statistical analysis reviewed and modified the questionnaire and drafted the manuscript. HAW conceived the draft of the interview, performed the pretest and did the sampling process. TH, BtW and US participated in the study design and helped to draft the manuscript. SB and DB conceived the study. All authors read and approved the final manuscript.

## Pre-publication history

The pre-publication history for this paper can be accessed here:

http://www.biomedcentral.com/1471-2458/10/735/prepub

## Supplementary Material

Additional file 1 **Khat questionnaire**. Questionnaire for the structured interview concerning specific opinions about khat and its impact on subjective and public health, and on social and community functioning. Click here for file
