# Supplementary material for: Motives for khat use and abstinence in Yemen - a gender perspective
Source: BMC Public Health. 2010 Nov 27;10:735. doi: 10.1186/1471-2458-10-735 (PMC3006386; doi:10.1186/1471-2458-10-735)
Supplement: Additional file 1 — Khat questionnaire. Questionnaire for the structured interview concerning specific opinions about khat and its impact on subjective and public health, and on social and community functioning. [file 1471-2458-10-735-S1.DOC]

Interviewer information!

The items A to J are always to be collected orally!

Personal information:

A - Gender 🞏 female 🞏 male

B1 - Does the subject occasionally use khat? 🞏 yes 🞏 no

*Interviewer instruction: Check yes, if khat was used at least once during the last year.*

B2 - Did the subject use khat during the last month? 🞏 yes 🞏 no

*Interviewer instruction: Continue interview only, if answers to items B1 and B2 are either yes/yes or no/no.*

C - Marital status 🞏 single 🞏 married 🞏 divorced 🞏 widowed

D - Age __ __ years

E - Education 🞏 attended no school or primary school only

🞏 attended secondary school

🞏 had vocational training or went to college

🞏 unknown/no response

F - Occupation 🞏 full time 🞏 home maker

🞏 part time 🞏 no work

🞏 other 🞏 unknown / no response

G - Primary source of income

🞏 own work 🞏 supported by relatives

🞏 pension 🞏 own assets

🞏 welfare 🞏 other

🞏 unknown / no response

H - How often do you use khat? 🞏 daily 🞏 at least weekly 🞏 less than weekly

I - On days of khat use: For how many hours do you consume khat? ______ h

J - For how many years have you been using khat? ______ yrs

Dear Madam! Dear Sir!

You are requested to participate in a scientific interview about concerning personal beliefs about khat use. With your help we are trying to gain a better understanding of the motives why people use khat or refrain from using khat. The whole interview will take about 30 to 40 minutes of your time. Your participation is entirely voluntary. You may abort the interview at any time. The survey is anonymous, but it is intended that your responses are analysed statistically. Any data published from this study will be in such a form to ensure that no participant can be identified by the reader. Your help is greatly appreciated.

If you agree to this, you are asked to read the following questionnaire thoroughly and answer the questions as correctly as possible. Please answer “Yes”, “Partly” or “No” depending on whether you believe fully, partly or don’t believe the corresponding statement. It is possible that you cannot answer some questions, because you are unsure or you have no knowledge or opinion about the matter. Please answer “No opinion”, if that is the case. Your interviewer will be with you during the interview to answer any questions you may have.

Hussein Al-Warith

Sample item:

Do you believe that …

| khat causes loss of appetite? | √Yes |  | Partly |  | No |  | No opinion |
| --- | --- | --- | --- | --- | --- | --- | --- |

This is the way you would mark the item, if you agreed fully with the belief in question.

Please check only one of the four possible answers for each of the questions.

Please do not write your name or any other identifying information on the questionnaire.

Do you believe that …

| 1 | The partner's ability to take care of the family is impaired by consuming khat? | Yes |  | Partly |  | No |  | No opinion |
| --- | --- | --- | --- | --- | --- | --- | --- | --- |
| 2 | consuming khat leads to relief of social and mental stress? | Yes |  | Partly |  | No |  | No opinion |
| 3 | consuming khat leads to a loss of esteem in the family? | Yes |  | Partly |  | No |  | No opinion |
| 4 | khat leads to an unhealthy complexion? | Yes |  | Partly |  | No |  | No opinion |
| 5 | khat rituals are the best way to spend one's time? | Yes |  | Partly |  | No |  | No opinion |
| 6 | khat is harmful to the unborn child? | Yes |  | Partly |  | No |  | No opinion |
| 7 | khat leads to a change in psyche and behaviour? | Yes |  | Partly |  | No |  | No opinion |
| 8 | khat causes moodyness? | Yes |  | Partly |  | No |  | No opinion |
| 9 | khat consumption causes agitation and anxiousness? | Yes |  | Partly |  | No |  | No opinion |
| 10 | khat is stimulating? | Yes |  | Partly |  | No |  | No opinion |
| 11 | khat is a drug? | Yes |  | Partly |  | No |  | No opinion |
| 12 | the family as a whole suffers financially if khat is consumed by the adults? | Yes |  | Partly |  | No |  | No opinion |
| 13 | khat consumption is the cause of social problems? | Yes |  | Partly |  | No |  | No opinion |
| 14 | khat consumption is a bad habit and should be eliminated? | Yes |  | Partly |  | No |  | No opinion |
| 15 | women should not consume khat, because this is especially bad for the family? | Yes |  | Partly |  | No |  | No opinion |
| 16 | khat consumers have a poor physical appearance? | Yes |  | Partly |  | No |  | No opinion |
| 17 | khat causes partners to fall out with each other? | Yes |  | Partly |  | No |  | No opinion |
| 18 | khat is addictive? | Yes |  | Partly |  | No |  | No opinion |
| 19 | khat improves the experience during sexual intercourse? | Yes |  | Partly |  | No |  | No opinion |
| 20 | khat consumption leads to loss of valuable work time? | Yes |  | Partly |  | No |  | No opinion |
| 21 | children of khat consuming parents do worse in school? | Yes |  | Partly |  | No |  | No opinion |
| 22 | khat causes poverty and squalor in Yemeni society? | Yes |  | Partly |  | No |  | No opinion |
| 23 | khat consumption is a factor in divorces? | Yes |  | Partly |  | No |  | No opinion |
| 24 | khat consumption is harmful to people's esteem and sophistication? | Yes |  | Partly |  | No |  | No opinion |
| 25 | khat consumption impairs the relationships between parents and their children? | Yes |  | Partly |  | No |  | No opinion |
| 26 | khat causes constant aggression between spouses? | Yes |  | Partly |  | No |  | No opinion |
| 27 | khat consumption causes lack of food in the family? | Yes |  | Partly |  | No |  | No opinion |
| 28 | khat consumption of parents leads to conduct disorders in their children? | Yes |  | Partly |  | No |  | No opinion |
| 29 | khat consumption improves relationships in our society? | Yes |  | Partly |  | No |  | No opinion |
| 30 | khat is a reason for most family problems? | Yes |  | Partly |  | No |  | No opinion |
| 31 | khat consumption by mothers causes neglect of their children and the household? | Yes |  | Partly |  | No |  | No opinion |
| 32 | khat rituals solve many social problems? | Yes |  | Partly |  | No |  | No opinion |
| 33 | khat consumption causes a higher crime rate? | Yes |  | Partly |  | No |  | No opinion |
| 34 | khat consumption prevents the use of other (illicit) drugs? | Yes |  | Partly |  | No |  | No opinion |
| 35 | khat causes depression? | Yes |  | Partly |  | No |  | No opinion |
| 36 | khat makes you attractively slim? | Yes |  | Partly |  | No |  | No opinion |
| 37 | khat improves physical fitness? | Yes |  | Partly |  | No |  | No opinion |
| 38 | khat rituals cause back injuries through long periods of sitting? | Yes |  | Partly |  | No |  | No opinion |
| 39 | khat causes constipation? | Yes |  | Partly |  | No |  | No opinion |
| 40 | khat causes loss of appetite? | Yes |  | Partly |  | No |  | No opinion |
| 41 | khat consumers are undernourished? | Yes |  | Partly |  | No |  | No opinion |
| 42 | khat consumption causes haemorrhoids? | Yes |  | Partly |  | No |  | No opinion |
| 43 | khat causes restlessness and nervousness? | Yes |  | Partly |  | No |  | No opinion |
| 44 | khat consumption impairs the function of the jaw muscles? | Yes |  | Partly |  | No |  | No opinion |
| 45 | khat improves memory retentiveness and concentration? | Yes |  | Partly |  | No |  | No opinion |
| 46 | khat houses are a source of epidemic infections? | Yes |  | Partly |  | No |  | No opinion |
| 47 | khat consumption is bad for one's health? | Yes |  | Partly |  | No |  | No opinion |
| 48 | khat worsens oral hygiene? | Yes |  | Partly |  | No |  | No opinion |
| 49 | khat causes impaired mood? | Yes |  | Partly |  | No |  | No opinion |
| 50 | you feel well and relaxed while consuming khat? | Yes |  | Partly |  | No |  | No opinion |
| 51 | khat consumers wake up tired the next morning? | Yes |  | Partly |  | No |  | No opinion |
| 52 | khat causes high blood pressure? | Yes |  | Partly |  | No |  | No opinion |
| 53 | khat causes low blood sugar? | Yes |  | Partly |  | No |  | No opinion |
| 54 | khat causes a reduced need for sleep? | Yes |  | Partly |  | No |  | No opinion |
| 55 | khat causes panic attacks? | Yes |  | Partly |  | No |  | No opinion |
| 56 | khat causes loneliness? | Yes |  | Partly |  | No |  | No opinion |
| 57 | khat consumption at work causes laziness? | Yes |  | Partly |  | No |  | No opinion |

Thank you very much!
